# Vehicle Recognition and Driving Information Detection with UAV Video Based on Improved YOLOv5-DeepSORT Algorithm

**DOI:** 10.3390/s25092788

**Published:** 2025-04-28

**Authors:** Binshuang Zheng, Jing Zhou, Zhengqiang Hong, Junyao Tang, Xiaoming Huang

**Affiliations:** 1 Research and Development Center of Transport Industry of New Generation of Artificial Intelligence Technology, Zhejiang Scientific Research Institute of Transport, No. 705 Dalongjuwu Rd., Hangzhou 311305, China; zhengbs@njupt.edu.cn; 2 School of Modern Posts, Nanjing University of Posts and Telecommunications, Nanjing 210023, China; b22110208@njupt.edu.cn; 3 China State Construction Engineering (Hong Kong) Limited, Hong Kong 999077, China; 4 School of Transportation, Southeast University, Nanjing 211189, China; huangxm@seu.edu.cn; 5 National Demonstration Center for Experimental Education of Road and Traffic Engineering, Southeast University, Nanjing 211189, China

**Keywords:** ramp, UAV video, vehicle recognition, YOLOv5 algorithm, vehicle track information

## Abstract

To investigate whether the skid resistance of the ramp meets the requirements of vehicle driving safety and stability, the simulation using the ideal driver model is inaccurate. Therefore, considering the driver’s driving habits, this paper proposes the use of Unmanned aerial vehicles (UAVs) for the collection and extraction of vehicle driving information. To process the collected UAV video, the Google Collaboration platform is used to modify and compile the “You Only Look Once” version 5 (YOLOv5) algorithm with Python 3.7.12, and YOLOv5 is retrained with the captured video. The results show that the precision rate P and recall rate R have satisfactory results with an F1 value of 0.86, reflecting a good P-R relationship. The loss function also stabilized at a very low level after 70 training epochs. Then, the trained YOLOv5 is used to replace the Faster R-CNN detector in the DeepSORT algorithm to improve the detection accuracy and speed and extract the vehicle driving information from the perspective of UAV. By coding, the coordinate information of the vehicle trajectory is extracted, the trajectory is smoothed, and the frame difference method is used to calculate the real-time speed information, which is convenient for the establishment of a real driver model.

## 1. Introduction

There are two reasons for the frequent accidents on the ramp: poor road conditions (objective factors) and the driver’s psychological state (subjective factors). Among them, the driver’s driving behavior is affected by psychological subjective factors, and there are obvious individual differences. To study the specific reactions of vehicles and drivers during driving, one method is to hire a large number of drivers to drive in the field, and the other method is to employ drivers to drive with simulation driving software. The enforceability of both methods is not high. For the first method, drivers tend to prefer safer driving behaviors because they know in advance that they will participate in the experiment and cannot restore the most widespread driving behaviors. The second method cannot accurately restore the real road conditions, so it is considered that a large-scale driving test is not feasible. Traditional methods of vehicle driving information extraction include monitoring equipment, vehicle sensors, satellite remote sensing cameras, and GPS positioning [1,2,3]. However, these methods have their shortcomings: monitoring equipment such as Closed-Circuit Television (CCTV) cameras have limited shooting positions and angles and a low degree of observation freedom. At a transportation facility, its presence or flashing light during shooting may certainly affect driver psychology [4]. While vehicle sensors are theoretically possible for large-scale road traffic applications with the help of technologies such as 5G, there are a number of challenges to putting them into practice. Not only does it require solving technical adaptation challenges, but it will also require significant regulatory changes; thus, at this stage, vehicle sensors can still only be used in a small number of vehicles. Satellite remote sensing cameras can only operate in a fixed orbit, making continuous observation difficult [5]. GPS is generally used as an aid to collect other information. GBAS-based GNSS and 5G, if mandated in all cars, would provide the best solution, but this could result in greater costs and require complex technology, which is why most western democracies are likely to be hesitant. Therefore, the use of UAVs for the collection of vehicle driving information is being gradually adopted by researchers [6,7,8,9].

UAV, as the potentially cutting-edge method for studying traffic flow and behavior at present, is characterized by safety, efficiency, speed, accuracy, etc. It can accurately collect information on vehicle tracks, speed, etc. without affecting driving behavior [10]. Therefore, it has been gradually used in the field of traffic data mining by researchers and combined with analytical software to simulate driving behavior [11,12,13]. Earlier, UAVs were used in traffic research by Coifman et al. [14,15], Agrawal et al. [16], and Ruhe et al. [17], who began to try using UAVs to collect traffic volume for traffic analysis in the early 21st century. Since then, more researchers have tried using UAVs for transportation-related research. After extracting the vehicle driving information with UAVs and successfully performing the skid resistance analysis, improving the skid resistance of the road surface is the key to ensuring driving safety.

The idea of vehicle recognition with UAV video can be divided into two categories [18]: one is to extract the motion region in the video first and then further judge the vehicle target; the other is to perform machine learning based on the existing vehicle’s appearance features first and obtain the candidate samples by searching the video sequences to be detected, then calculate the matching degree of the candidate samples one by one and complete the classification of the samples by applying the classifiers built by the previous training, so as to achieve the purpose of monitoring. The filter method and optical flow method are two relatively mainstream methods.

The filter method is one of the earliest applied methods, containing three parts: the state equation, observation equation, and posteriori density, which transforms the target problem into a probability density estimation problem. According to the Bayesian principle, the prior probability density function information and the observation probability density function information can calculate the current posteriori probability density of the target, while the target tracking algorithm assumes that the state equation and observation equation of the target are linear Gaussian processes. Therefore, the posteriori probability density of the system state obeys the Gaussian distribution [19]. Accordingly, based on the above, the Kalman filter can be applied to video vehicle information collection [20]. In the subsequent applications, researchers found that the posteriori probability is not necessarily linear, so the particle filter method was proposed [21]. After UAV traffic monitoring was applied, both filter methods were applied and optimized. In 2005, Rad et al. [22] proposed a moving target indication and tracking system using Kalman filter for vehicle detection. This method can perform morphous recognition and contour extraction with 96% accuracy, but the measurement speed is only 11 frames per second. Since then, more and more researchers have used the Kalman filter or particle filter to monitor vehicles in videos. For example, Jones et al. [23] used the particle filter for motion compensation of the sensors between the motion frames detected in each frame through feature tracking, random sampling, consistency algorithm, and affine transformation. Fang et al. [24] used the particle filter and MeanShift algorithm to monitor vehicles and optimized the method. This method can automatically adapt to changes in vehicle size. Nair et al. [25] used the Kalman filter to monitor vehicles and validated the model with signal-to-noise ratio-normalized mean square error plots. Fernández-Sanjurjo et al. [26] achieved the simultaneous real-time process of more than 400 vehicles’ information, based on deep learning detectors for vehicle recognition and combined relevant filters and the Kalman filter for vehicle monitoring and tracking.

The concept of optical flow was first proposed by Gibson in 1950 [27]. This term describes the information that the object’s motion presents as a continuous change when observed by the human eye, known as the optical flow; the projection of a motion object onto a two-dimensional image presents the optical flow field of that motion object [28]. The earliest optical flow field computation was proposed by Horn et al. in 1980, providing important methods for the study such as 3D velocity field estimation and object motion analysis [29]. Traditional vehicle recognition and monitoring are based on stationary videography. It judges the motion objects by the difference between consecutive frames and then establishes the operation to reflect the moving trajectory of the motion target. The benefit of this method is its good applicability in fixed shooting conditions such as monitoring cameras. However, when UAV cameras are used to extract vehicle information, the background may also change due to UAV flight. Therefore, traditional patterns and mathematical models cannot solve the problem. In 1994, Barron et al. [30] proposed the optical flow method to solve the problem. After that, researchers have successively proposed computational methods such as the Horn–Schunck (HS) optical flow method and Lucas–Kanade (LK) optical flow method, among which the LK optical flow method is more applied in vehicle monitoring, and the Kanade–Lucas–Tomasi (KLT) corner detection method is derived from it.

There are many algorithms related to machine vision that are generated based on the filter and optical flow methods and have been applied in vehicle driving information extraction, such as the OpenCV-based MeanShift algorithm [24] and the CamShift algorithm [31] derived from it, the ViBe algorithm [18], and the sample-consistent background modeling algorithm [32]. Vehicle recognition methods with UAV video based on one-stage detector and two-stage detector methods have also been gradually increasing in recent years. For example, Peng et al. [33,34] successively performed vehicle recognition with UAV video based on deep learning and neural networks, respectively proposed an improved AlexNet model and an improved Faster R-CNN model, and applied them to vehicle recognition with UAV video, which showed a certain increase in accuracy and a reduction in training time compared to the use of the traditional model. Kim [35] performed image segmentation on UAV video based on deep learning to detect vehicle status while accurately recognizing traffic scenes and facilities and also accurately locate the incident location if an accident occurs. Chen et al. [36] used artificial intelligence chips to build lightweight neural networks to achieve automatic detection of video vehicle targets on the airborne end of the UAV. The results show that the network built by them effectively improves the detection accuracy and achieves a detection speed of 125.3 ms per frame while reducing the use of storage and ensuring light weight. Liu et al. [37] combined R-CNN with the Kalman filter, MeanShift-based algorithm, and online boosting tracking method, etc., and found that combining R-CNN with the Kalman filter has relatively less computation and higher accuracy in the application of a traffic scene with only dozens of objects.

The traditional idea of target detection is to regard the problem as a classification problem, where the target is first extracted and then classified using a classifier, i.e., “two-stage method”, followed by post-processing. Therefore, classifiers are in the state of reuse in the previous methods. Among the existing methods, the Deformable Parts Models (DPM) method adopts the approach of sliding window for brute force traversal, causing the reuse of classifiers [38], while methods such as R-CNN [37] and Fast R-CNN [33,34] also extract potential candidate frames and then classify all of the extracted information one by one with neural networks. The YOLO algorithm was proposed by J. Redmon et al. in 2016, and the first version was YOLOv1. Unlike traditional methods that reuse classifiers, YOLO adopts an integrated algorithm, which transforms the target detection problem into a regression problem, and outputs the results with only a single forward inference, i.e., “one-stage method” [39]. The YOLO algorithm has many advantages, particularly in UAV-based applications where real-time performance and adaptability to aerial perspectives are critical [11,40]. First of all, the traditional two-stage method is less efficient; even the more powerful Fast R-CNN can only achieve seven frames per second of image processing, while YOLO can achieve a processing efficiency of 45 frames per second, and the faster Fast YOLO can even achieve 150 frames, which is crucial for real-time performance. Secondly, each step in the two-stage method needs to operate independently, but its results will directly affect the overall output; so, once a problem occurs in one part of the process, the whole detection result will be wrong. YOLO uses only one neural network, which can directly output the coordinate information of multiple objects from the image at one time. Thirdly, the traditional methods can only detect the selected specific region, which leads to certain errors in its judgment of the relationship between objects, and the background may be judged as a specific object. The YOLO algorithm can directly analyze the image at full scale, which can greatly decrease the misjudgment rate of the background from a global perspective and can clearly judge the interaction relationship between objects. Furthermore, YOLO is more generalizable; i.e., the materials used in the two stages of training and prediction can come from different environments, thus adapting well to target detection in different environments.

In summary, the basic development process of vehicle information extraction based on drone video is from first identifying a single vehicle to gradually developing to simultaneously identifying multiple vehicles. Moreover, in this process, the frame rate increases, the detection time is shortened, the number of vehicles can be identified at the same time, and the noise and other problems are gradually solved. In this development process, the most important point is to shift from the extraction of existing videos to real-time extraction and analysis, reflecting “real-time”, which can greatly help timely traffic control decisions. In order to study whether the anti-skid performance of the ramp meets the requirements of vehicle safety and stability, it is not accurate to use the ideal driver model for simulation. The real driver behavior must be collected instead of the ideal driver model to reflect the impact of the design elements of the ramp on the driver’s judgment. In order to achieve this goal, the UAV is used to collect vehicle driving information, and the driving behavior is obtained through video processing. In view of this, this paper proposes the use of UAVs for collecting and extracting vehicle driving information based on the YOLO algorithm. To avoid the inaccuracy of the YOLO algorithm for aerial video recognition, the on-ramp vehicle driving video collected by UAV is used for labeling firstly. Meanwhile, a training set containing a large amount of material is built, and the Google Collaboration platform is called upon to optimize the YOLO algorithm under the PyTorch kernel and retrain it with the captured video. Then, the trained YOLOv5 is used to replace the Faster R-CNN detector in the DeepSORT algorithm to improve the detection accuracy and speed and extract the vehicle driving information. Finally, the coordinate transformation from the perspective of UAV is calculated.

## 2. Basic Principle of YOLO Algorithm

The full name of the YOLO algorithm is “You Only Look Once”, and the first version, YOLOv1, was proposed by Redmon et al. in 2016. Unlike traditional methods that reuse classifiers, YOLO adopts an integrated algorithm, which transforms the target detection problem into a regression problem, and outputs the results with only a single forward inference, i.e., “one-stage method”, hence its name, “You Only Look Once” [39].

### 2.1. Training of YOLO Algorithm

Like all computer vision algorithms, the training of the YOLO algorithm also relies on manual labeling. The main process is to compare the results predicted by the algorithm with the results of manual labeling and minimize the error [41]. When the training image is input to the algorithm, the algorithm will first automatically divide the image into several grid cells. If the center of the object falls in a grid cell, that grid cell is responsible for recognizing and detecting the object. Each grid cell randomly generates a number of rectangular bounding boxes, and every bounding box is fitted to manually labeled objects, with five parameters output, namely, the relative position between the center of the bounding box and the grid cell (x, y), the relative width and length of the predicted image (w, h), and the confidence that the algorithm assigns to each of the bounding boxes. Among all the bounding boxes, the one with the largest intersection over union (IoU) with the manually labeled images will be given the maximum confidence. The confidence is calculated as

(1) $$Pr\left(Object\right)\times {IoU}_{pred}^{truth},$$

where Pr(Object) is the parameter to judge whether the bounding box contains an object or not (1 if yes, 0 if no); ${IoU}_{pred}^{truth}$ is the IoU of the predicted image and the manually labeled image with the higher confidence, the thicker contour of the bounding box, and the higher probability of obtaining retention at the end.

For the five parameters, x, y, w, h, and confidence, the loss function is the basis for assessing the accuracy of detection. In this case, YOLOv1 uses the sum-of-squares error as the loss function, and the five parameters are represented in the loss function as

(2) $$\lambda_{coord}\sum_{i=0}^{S^{2}}\sum_{j=0}^{B}{ℾ}_{ij}^{obj}[{\left(x_{i}-\hat{x_{i}}\right)}^{2}+{\left(y_{i}-\hat{y_{i}}\right)}^{2}]\;\;+\lambda_{coord}\sum_{i=0}^{S^{2}}\sum_{j=0}^{B}{ℾ}_{ij}^{obj}[{\left(\sqrt{w_{i}}-\sqrt{\hat{w_{i}}}\right)}^{2}+{\left(\sqrt{h_{i}}-\sqrt{\hat{h}}\right)}^{2}]\;\;+\sum_{i=0}^{S^{2}}\sum_{j=0}^{B}{ℾ}_{ij}^{obj}{\left(C_{i}-\hat{C_{i}}\right)}^{2}\;\;+\lambda_{noobj}\sum_{i=0}^{S^{2}}\sum_{j=0}^{B}{ℾ}_{ij}^{noobj}{\left(C_{i}-\hat{C_{i}}\right)}^{2}\;\;+\sum_{i=0}^{S^{2}}{ℾ}_{i}^{obj}\sum_{c\in classes}{\left(p_{i}(c)-\hat{p_{i}}(c)\right)}^{2}.$$

The YOLOv1 algorithm initially used the 1000-category dataset of ImageNet [1].

### 2.2. Detection Principle of YOLO Algorithm

The basic detection principle is the same as the training stage. It also needs to judge two values of $Pr\left(Object\right)$ and ${IoU}_{pred}^{truth}$ but only performs regression calculations without direct output. At the detection stage, each grid cell, in addition to generating the bounding boxes to determine whether there is an object in a certain region, has to perform object identification, and the algorithm needs to judge the specific attributes of the object based on the learning that has been learned from previous training. The algorithm compares the features of all trained objects with the target object, outputs the respective conditional probabilities $Pr({Class}_{i}|Object)$ for each object, and multiplies them with confidence to obtain the respective probabilities for each class of objects:

(3) $$Pr\left({Class}_{i}|Object\right)\times Pr\left(Object\right)\times {IoU}_{pred}^{truth}=Pr\left({Class}_{i}\right).$$

**Figure 1:**
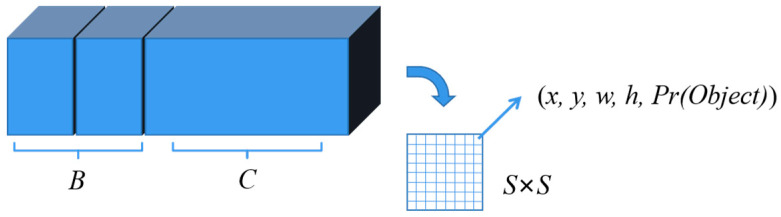
YOLOv1 algorithm output structure composition [41].

Assuming that the image is divided into S×S grid cells, each of which produces *B* bounding boxes, and there are C categories of objects, the detection will be encoded into a tensor of size S×S×(B×5+C), as shown in Figure 1. In YOLOv1, S=7, B=2, C=20; thus, there are a total of 98 bounding boxes, and the dimension of each grid vector is 30.

The design of the grid cells is such that each grid can only perform detection within a limited area, which may also lead to larger objects being detected by multiple grid cells. Therefore, it is necessary to introduce non-maximal suppression to filter the bounding boxes with lower confidence.

The YOLO algorithm, a classic algorithm in target detection, is famous for its high speed, high efficiency, and higher accuracy. YOLO has evolved several versions from V1 to the latest version, each of which has been improved to increase detection accuracy and speed to adapt to different application scenarios. The pros and cons of the different algorithms are compared, as shown in Table 1 below. Overall, YOLOv5 has become the most widely used version of the YOLO series due to its friendly development, excellent performance, and rich community resources. Importantly, YOLOv5 can run on architectures such as TensorFlow and PyTorch 1.8.0, which means that it is free from the original Darknet backbone and can be integrated with a more general backbone. Therefore, this paper will use the improved YOLOv5 algorithm for vehicle information recognition.

## 3. Labeling and Training of Vehicles with UAV Video

### 3.1. Labeling of Vehicles with UAV Video

**Figure 2:**
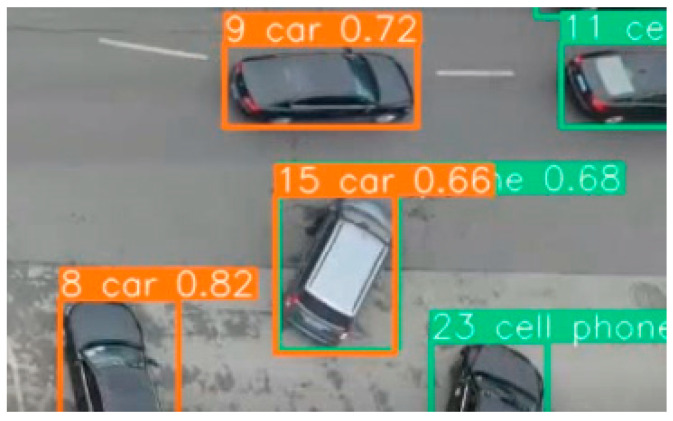
Misjudgment generated by the training set of YOLO itself [51].

Since the training of YOLOv5 (hereinafter referred to as YOLO) is based on the MS COCO dataset, while the original dataset does not contain the vehicle data under the UAV perspective, some of the distinctive features reflecting the vehicle (such as wheels, rearview mirrors, and vehicle head, etc.) cannot be recognized, so some vehicles are recognized as cellphones with similar shapes and colors as the vehicles, as shown in Figure 2. In Figure 2, the vehicle numbered 15 is marked with two detection frames, “car” and “cellphone”, and both of their confidences reach more than 0.6, indicating that both interfere with computer vision. Therefore, the original “car” and “cellphone” categories in YOLO need to be selected and modified and then assisted in computer retraining through manual labeling. At this time, it is not necessary to retrain the entire neural network, but only to train a new network layer based on the vehicles in the basic training set with the vehicle top features, which can be adapted to vehicle recognition under unused background conditions [51]. This paper uses the UAV video collected from highway on-ramps in Nanjing for vehicle top-view perspective labeling. To enhance the training effect, the anti-interference ability of detection, and the adaptability to different environments, the videos used contain working conditions such as the same ramp during flat and peak hours, different ramps during the same period, and ramps with different weather conditions (sunny, cloudy, and rainy); meanwhile, videos of some parking lots and downtown traffic are introduced, which have high vehicle densities and are of great benefit in training YOLO small object recognition accuracy.

First, the captured UAV video is extracted with key frames. The frame rate of each video is not the same, but generally, it is 24~30 frames per second, so the key frame extraction is set to be performed every 24 frames. Create a new environment in Anaconda PowerShell, install OpenCV, and then use the “cv2.VideoCapture” function to extract key frames. Part of the code is as follows (Code 1):
**Code 1.** UAV video key frame extraction and storage1import cv22import os3# location of the video to be extracted4video_path=r”./UAVvid1.mp4”5# location of the image to be saved6img_path=r’./Images’7vidcap = cv2.VideoCapture(video_path)8(cap,frame)= vidcap.read()9while cap:10  cv2.imwrite(os.path.join(img_path,’%.6d.jpg’%count),frame)11  print(‘%.6d.jpg’%count)12  count += 1…


**Figure 3:**
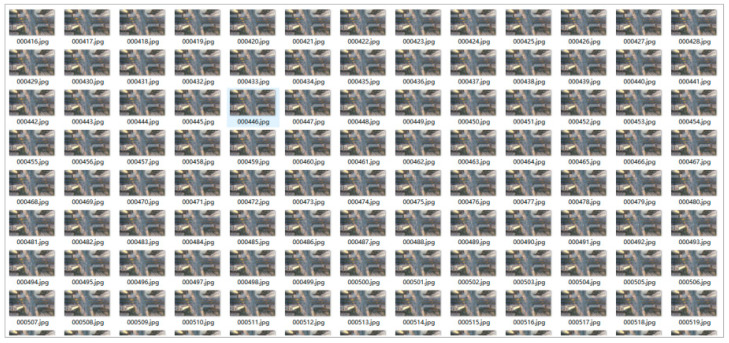
Samples of training and validation sets.

**Figure 4:**
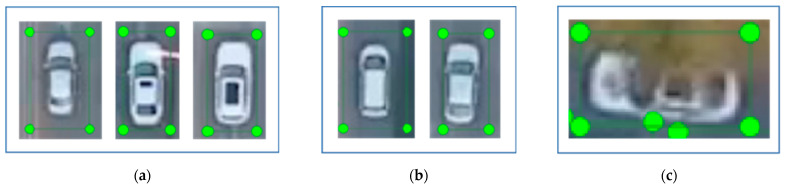
Different categories of vehicle labeling: (**a**) sunroof and sunroof-less vehicles; (**b**) hatchbacks (two-box) and three-box vehicles; (**c**) visible and partially obscured vehicles.

**Figure 5:**
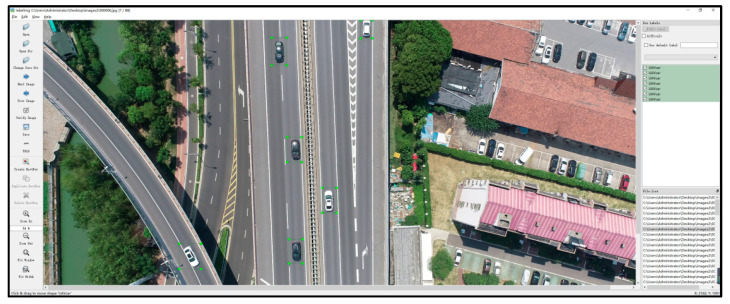
Labeling vehicles with UAV perspective using LabelImg.

A total of 782 valid images are extracted and used as the training set and validation set, as shown in Figure 3. The extracted images are labeled with LabelImg. YOLOv5 can directly recognize txt format labeling files without the using traditional xml format labeling files; therefore, it is able to switch the mode to “YOLO” and create the new categories “UAVcar” and “UAVbus” for use in this study. Three groups of vehicles are labeled according to the different features of small vehicles, including sunroof and sunroof-less vehicles (Figure 4a), hatchbacks (two-box) and three-box vehicles (Figure 4b), and visible and partially obscured vehicles (Figure 4c). Figure 5 shows the overall labeling, and the saved file contains “class.txt” as well as txt files named after the original image, which can be directly called by YOLOv5.

In the extracted images, about five to eight small cars are labeled in each one (totaling more than 5000 samples); buses and trucks are labeled according to whether they are contained in the images or not, and in the images containing large cars, they should be labeled as much as possible. After that, the pictures extracted from different scenarios are randomly disordered and assigned to the training, validation, and test sets in the ratio of 8:1:1, which are stored in three folders, namely, “train”, “val”, and “test”, under the “images” directory, and the labeling files of the corresponding images are stored in the corresponding folders of the “labels” directory. The call is performed using the following code (Code 2):
**Code 2.** Dataset partitioning and label configuration1train: ./data/yolo_UAV/images/train2val: ./data/yolo_UAV/images/val

### 3.2. Results of YOLO Training

The training of YOLO requires a certain computer configuration. Limited by the machine hardware, this study was conducted with Google Collaboration (Google LLC, Mountain View, CA, USA), calling the online server. The hardware assigned to the server is as follows: the GPU is Nvidia Tesla K80, the GPU memory is 12GB, and the CUDA version is 11.2. YOLOv5 is based on the PyTorch architecture, and to ensure the speed and stability of operation, the configuration of the main software versions is shown in Table 2.

**Figure 6:**
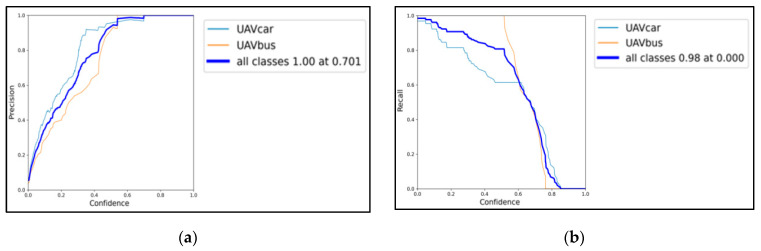

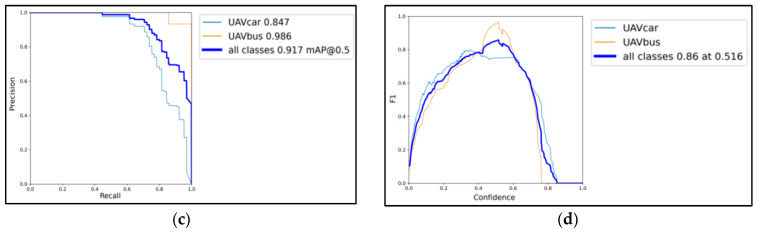
Results of YOLO training. (**a**) relationship between confidence and the precision rate; (**b**) relationship between confidence and the recall rate; (**c**) PR plots; (**d**) relationship between confidence and the F1 value.

**Figure 7:**
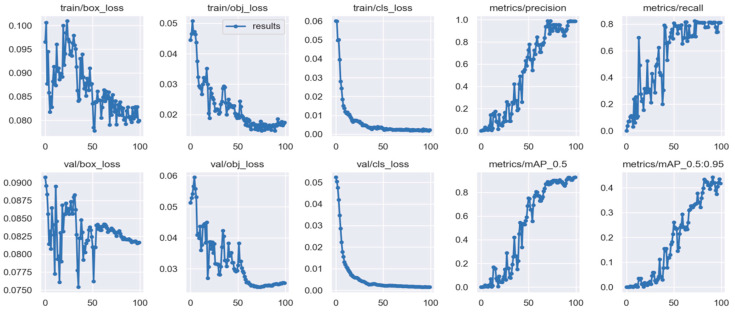
Relationship between the training epoch and loss ratio, mAP.

**Figure 8:**
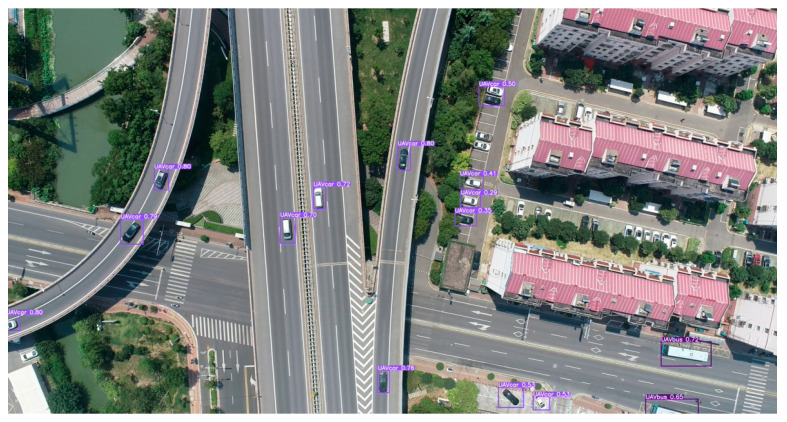
Actual detection results.

YOLO training is performed with the program “train.py”, calling the compiled “UAV_yolov5s.yaml” architecture and the corresponding “yolov5.pt” weights file, and saving the training results into folders. To perform training, the number of rounds (epoch) and the batch-size for each training need to be set. Set the epoch to 100 training rounds and the batch-size to 64. Perform training in a conda environment. During the run, the GPU memory usage was 10.4 G. The training eventually produced new weight files, and the results are shown in Figure 6, Figure 7 and Figure 8.

Figure 6a reflects the relationship between confidence and the precision rate P. For a given object category, P represents the ratio of the number of correct predictions over the total number, i.e., the percentage of correct predictions. There is a very significant increase in P as the confidence of the learning increases, and eventually, 100% accuracy is achieved for all categories of objects at a confidence of 0.701.

Figure 6b reflects the relationship between confidence and the recall rate R. For a given object category, R represents the ratio of the number of correct predictions over the true total, i.e., the percentage of predicted objects found. Normally, the increase in confidence will lead to the decrease in R. It is noticed that R eventually decreases to 0 at the confidence of 0.98 for all categories of objects, and for the dominant small cars, it also decreases to 0 at the confidence of more than 0.9, indicating that the learned YOLO has a high recognition success for small cars. For large vehicles, R decreases to 0 at the confidence of more than 0.75, which is caused by too few samples and inconspicuous aerial photo features of large vehicles, but YOLO still has a high recognition accuracy.

Figure 6c reflects the relationship between recall and precision, i.e., PR plots, where the R value is generally the horizontal coordinate, and the P value is the vertical coordinate. As R increases, the number of objects of a certain category that are predicted increases; i.e., those with a lower probability are gradually predicted. An excellent PR relationship is measured by the area; i.e., the larger the area at the bottom left of the PR plot, the better the model works for that dataset. It is observed that the YOLO model is fairly effective in recognizing vehicles from aerial videos.

Figure 6d reflects the relationship between confidence and the F1 value. From previous conclusions, it is obvious that the P value and the R value are naturally contradictory, so the F1 value is introduced. The F1 value is a harmonic mean of the P and R values, and its function is to calculate a harmonic mean of them, which can better reflect the actual average of the two. It can be calculated by Equation (4) and (5):

(4) $$f_{1i}=2\times \frac{R_{i}\times P_{i}}{R_{i}+P_{i}},$$

(5) $$F_{1}={\left(\frac{1}{n}\sum_{i=0}^{n-1}f_{1i}\right)}^{2}.$$

The intersection with the PR curve is called the equilibrium point by introducing a diagonal line with slope 1 from the origin of Figure 6c. The equilibrium point generally determines the area enclosed by the PR curve; i.e., different PR relationships will have different equilibrium points. The F1 value considers both the P and R values, allowing both to be maximized and balanced simultaneously. As shown in Figure 6d, the model overall reaches the maximum F1 value of 0.86 at the confidence of 0.516, balancing the PR relationship very well.

Figure 7 reflects the change of training indexes with the training epoch. The first three columns show the loss function. It is seen that the loss in both the training and validation sets decreases significantly with the increase of training epochs, and the loss function reaches a stable and low level after 70 training epochs.

The two more important indexes are mAP@0.5 and mAP@[0.5:0.95], where mAP is the mean average precision, which is calculated by averaging the AP again. The mAP0.5 represents the mean AP value in the case where the detected IoU threshold is set at 0.5, while mAP@[0.5:0.95] represents the mean AP value when the IoU threshold is set from 0.5 to 0.95 in steps of 0.05. The two images in the fourth and fifth columns of the second row in Figure 7 reflect these two parameters. It can be seen that after 70 training epochs, the overall mAP@0.5 can reach more than 90%, and mAP@[0.5:0.95] can reach more than 40%, showing higher recognition performance.

### 3.3. Field Test

The “detect.py” file is used for trained YOLO detection, and the optimal weight file “best.pt” generated after training is called during detection. Figure 8 shows the video detection using the trained program, with a randomly exported frame, in which a total of 13 small vehicles and two large vehicles are detected. It is observed that the program in the real case has some detection error only for vehicles at densely obscured places (e.g., the vehicle obscured by trees near the house on the right side in Figure 8) and has high detection accuracy for vehicles that are less obscured and are actually involved in the traffic during the action.

## 4. Vehicle Information Extraction Based on Improved YOLOv5-DeepSORT

### 4.1. Improvement of DeepSORT Algorithm

**Figure 9:**
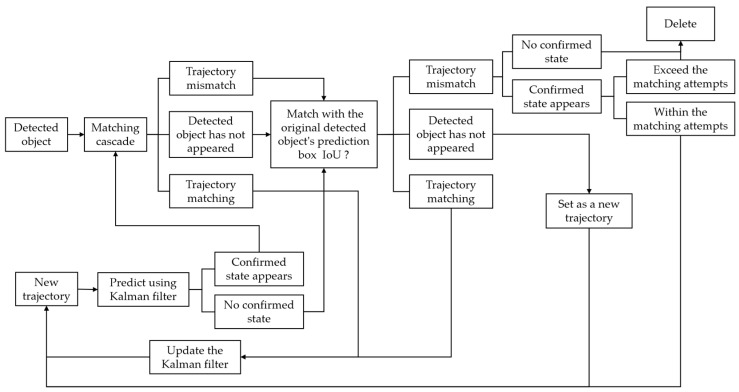
Basic principle schematic of DeepSORT.

The overall framework of DeepSORT (as shown in Figure 9) is inherited from the SORT algorithm, which retains the advantages of the Kalman filter and Hungarian algorithm, adds a matching cascade based on them, and incorporates the determination of confirmed state for the new trajectory, which is categorized as confirmed and unconfirmed [52]. DeepSORT contains a main program, “deep_sort.py”, and eight subroutines, including the detector “detection.py”, IoU comparison operator “iou_matching.py”, Kalman filter “kalman_filter.py”, and the track subroutine “track.py”, etc. Although the merit of the detector does not directly affect the quality of tracking, the accurate target detection can enhance tracking efficiency and reduce the error rate. The native SORT and DeepSORT use Faster R-CNN for target detection, whereas the YOLOv5-DeepSORT method used in this study replaces the original Faster R-CNN with the trained YOLOv5 for target detection.

To replace “detection.py” with YOLOv5, it is necessary to modify “deep_sort.py” and “track.py”, two programs involved in target recognition. First, the target track subroutine “track.py” is modified, and part of the code is as follows (Code 3):
**Code 3.** YOLOv5-compatible target tracking module…
1self.yolo_bbox = [0, 0, 0, 0]…
2def get_yolo_pred(self):3  return self.yolo_bbox…
4def update(self, kf, detection, class_id):5  self.yolo_bbox = detection…


The modified “track.py” initializes the bbox (bounding box) of YOLOv5, then defines the prediction function of YOLOv5 and assigns the detected objects to the bboxes, ensuring subsequent trajectory recognition using YOLOv5 as the detector.

After modifying “track.py”, it is necessary to update the part of the main program “detection.py” that calls “track.py”, so that the new detector can be used by the entire program at runtime. Part of the code is as follows (Code 4):
**Code 4.** Main program tracking logic update…
1def update(self, bbox_xywh, confidences, classes, ori_img, use_yolo_preds=True):2  self.height, self.width = ori_img.shape[:2]…
3for track in self.tracker.tracks:4  if not track.is_confirmed() or track.time_since_update > 1:5    continue6  if use_yolo_preds:7    det = track.get_yolo_pred()8    x1, y1, x2, y2 = self._tlwh_to_xyxy(det.tlwh)9  else:10    box = track.to_tlwh()11  x1, y1, x2, y2 = self._tlwh_to_xyxy(box)12  track_id = track.track_id13  class_id = track.class_id14  outputs.append(np.array([x1, y1, x2, y2, track_id, class_id], dtype=np.int))…


The code “use_yolo_preds=True” implements the application of YOLOv5 in DeepSORT prediction. In the following code, the main program calls the “track.py” subroutine to create and update trajectories.

### 4.2. Vehicle Coordinate Transformation

After vehicle recognition and tracking, the track, speed, and other information of the vehicle need to be extracted for use as a real driver model instead of the ideal driver model in the simulation experiments, eliminating the influence of the driver’s subjective factors on the road surface skid resistance requirements. Before extracting the vehicle information, it is required to perform coordinate transformation on the video information to transform the pixel coordinate information into real coordinate information.

The coordinate transformation starts with the calibration of the UAV camera. Since this study uses aerial photography with a purely overhead view, the effect of the pitch angle on the camera calibration can be disregarded; also, this study ensures as much as possible that the ramp is at the center of the captured video, so the adverse effect of image distortion can be neglected.

**Figure 10:**
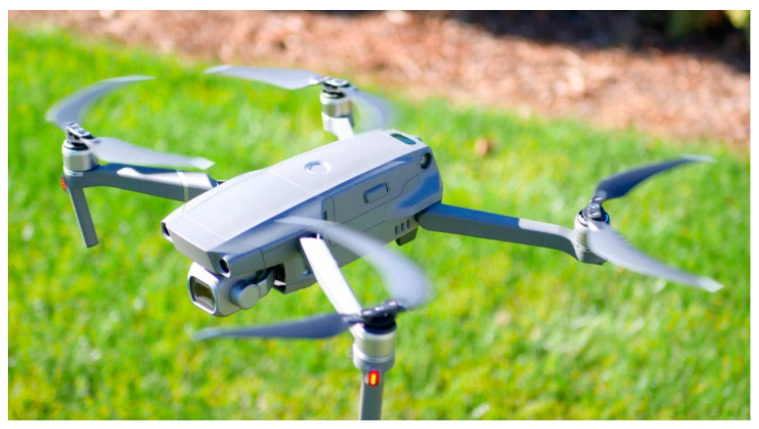
DJI Mavic 2 Pro.

The ground sample distance (GSD) is calculated based on the camera’s intrinsic parameters and the actual ground length. The GSD refers to the actual ground length represented by a single pixel in digital imagery. For instance, if the GSD in a video is 10 cm, this indicates that each pixel corresponds to 10 cm of actual ground length. The GSD is calculated using Equation (6):

(6) $$GSD=\frac{H\times a}{f},$$

where H is the relative flying height, a is the pixel size, and f is the lens focal length. The UAV used in this study is the DJI Mavic 2 Pro (DJI, Shenzhen, China), whose exterior is shown in Figure 10, with detailed specifications provided in Table 3.

From the parameters in Table 3, the focal length in Equation (6) is f=10; the pixel size a can be determined by the ratio of sensor size to image resolution: for 4K video boasting an image resolution of 3840 × 2160 pixels, the pixel size is calculated as $a_{1}=13.2\;mm/3840=3.4375\;\mu m$, $a_{2}=8.8\;mm/2160=4.07407\;\mu m$, and the corresponding pixel sizes need to be calculated according to the different image resolutions; for the relative flying height H, the built-in GPS can only provide the absolute height of the UAV, so the relative flying height H should be the absolute height of the UAV $H_{UAV}$ minus the actual road height $H_{road}$, which is obtained according to the design information.

Above all, Table 4 summarizes the calculated shooting reference. In the actual shooting, if the UAV is too close to the ground, the entire ramp may not be fully included in the picture; however, when the relative flying height is too large, it easily causes the vehicle to be too small, which is not conducive to recognition and detection of the algorithm. From Table 4, when the radius of captured ramp differs, the relative flying height can be selected according to the shooting width and shooting area. In general, to shoot the most common ramps with 40 km/h limits, a relative flying height of about 100 m is sufficient, and the DJI Mavic 2 Pro is able to achieve better results; when shooting ramps with 80 km/h limits (radius larger than 230 m), it is necessary to choose a relative flying height of 200 m or more, and it is appropriate to switch to the UAV equipped with a better camera for shooting in this case. After the shooting is completed and the GSD value is obtained, the recognized vehicle pixel coordinate and speed information are multiplied by this value to obtain the vehicle coordinate information in the real situation.

### 4.3. Vehicle Trajectory Extraction

Vehicle trajectory extraction is the most crucial step to reflect the driver’s judgment about a ramp in actual driving and obtain the real driver model. The vehicle trajectory information can be obtained by acquiring the position information of each vehicle target frame by frame.

#### 4.3.1. Vehicle Information Acquisition

In YOLOv5-DeepSORT, the center coordinates of each target vehicle and its ID value are derived, and the center coordinates of vehicles with the same ID are connected into a line, representing the vehicle trajectory; by calculating the position information of neighboring frames with the frame difference method, real-time speed information can be obtained. Use the following code to write and output the coordinates (Code 5):
**Code 5.** Vehicle trajectory center coordinate extraction1if len(outputs) > 0:2  for j, (output, conf) in enumerate(zip(outputs, confs)):3    bboxes = output[0:4]  # extract coordinate information4    id = output[4]  # extract vehicle ID information5    cls = output[5]  # extract vehicle class information…
6    if save_txt:7      x_center = output[0] / 2 + output[2] / 2  # calculate the x-coordinate of the bbox center8      y_center = output[0] / 2 + output[2] / 2  # calculate the y-coordinate of the bbox center9      with open(txt_path, ’a’) as f:10        f.write((‘%g ‘ * 10 + ‘\n’) % (frame_idx + 1, id, x_center, y_center,))  # write (frame number, ID, bbox center x, bbox center y)

With the above code, the one-to-one correspondence between the ID and coordinates of each detected vehicle is achieved, and the results will be saved in a txt format file.

#### 4.3.2. Vehicle Trajectory Drawing

The trajectory drawing of vehicle motion is achieved by connecting the center coordinates of each vehicle. To visually assess the accuracy of detection and tracking, real-time trajectory drawing is required to allow direct observation in the video; moreover, drawing the trajectory directly in the video also shows the exact position of the vehicle traveling in the lane and judges the driving behavior. Real-time trajectory drawing is achieved by writing the following code (Code 6):
**Code 6.** Real-time vehicle trajectory visualization1if len(outputs) > 0:2  for j, (output, conf) in enumerate(zip(outputs, confs)):3    bbox_xyxy = output[0:4]  # extract coordinate information4    id = output[4]  # extract vehicle ID information5    x_center = bbox_xyxy[0] /2 + bbox_xyxy[2] / 2  # calculate the x-coordinate of the bbox center6    y_center = bbox_xyxy[1] /2 + bbox_xyxy[3] / 2  # calculate the y-coordinate of the bbox center7    center = [x_center, y_center]8    dict_box.setdefault(id, []).append(center)  # call Python dictionary9  if frame_idx > 2:  # skip first 2 frames (DeepSORT can’t draw); start from frame 310    for key, value in dict_box.items():11      for a in range(len(value) − 1):12        color = COLORS_10[key % len(COLORS_10)]  # set trajectory color13        index_start = a14        index_end = index_start + 115        cv2.line(im0, tuple(map(int, value[index_start])), tuple(map(int, value[index_end])), color, thickness=2, lineType=8) # draw trajectory line

**Figure 11:**
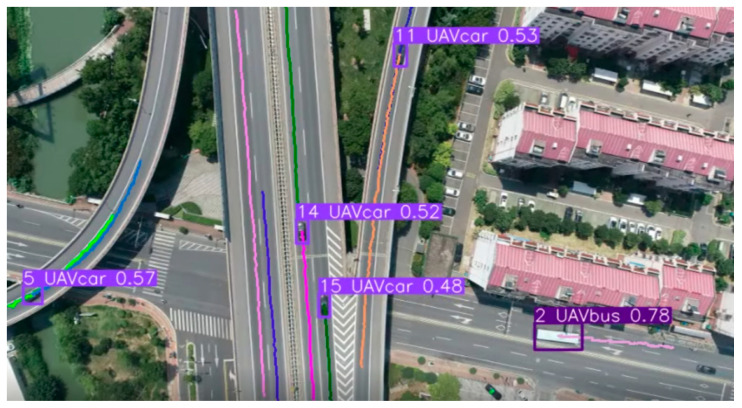
Vehicle trajectory drawing results.

After writing and running the above code, the trajectory line can be drawn in real time. The drawn trajectory lines are shown in Figure 10. In Figure 11, the trajectory lines show no mutation or interlacing, indicating that the detected and tracked trajectory lines of the program are usable, and the program can be used for vehicle trajectory extraction and drawing.

#### 4.3.3. Vehicle Trajectory Smoothing

Although the extracted vehicle trajectory is usable, it is somewhat inaccurate and even “jagged” when magnified several times because the coordinate points of each frame are connected by straight lines, and the size of the detection box of each frame is not exactly equal. Writing such a trajectory into CarSim as a real driver model will cause the driver to keep making steering adjustments, which easily leads to uncontrolled maneuvering. Therefore, it is necessary to smooth the trajectory according to the direction of the trajectory coordinates.

**Figure 12:**
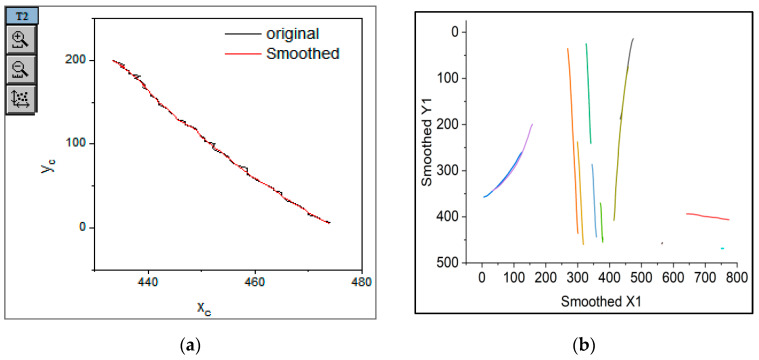
Vehicle trajectory after smoothing. (**a**) A vehicle trajectory; (**b**) Vehicle trajectories corresponding to Figure 11.

Import the coordinate file into Origin, navigate through “Analysis” to “Signal Processing” to “Smooth”, and apply Lowess (locally weighted regression) for smoothing. Set “span” to 0.1 to achieve better results, as shown in Figure 12. The colorful lines in Figure 12(b) correspond to the vehicle trajectories in Figure 11. 

## 5. Conclusions

To obtain the real driver behavior instead of the ideal driver model, this study adopts UAV to collect the vehicle driving information on the ramp. Firstly, the pros and cons of various versions of the YOLO algorithm are compared, choosing YOLOv5 as the version of target recognition detection algorithm in this study, and the basic principle of the YOLO algorithm is elaborated. Then, the Google Collaboration platform is further used to modify and compile the YOLOv5 algorithm with Python, and the captured videos are used for retraining, where over 5000 representative samples in different situations are trained for 100 cycles. The indexes, such as the P value, R value, and F1 value of the training, are analyzed, and the results of the study show the following:

(1) As the confidence of learning increases, there is a very significant increase in P, and eventually, a 100% precision rate is achieved for all categories of objects at the confidence of 0.701. There is a significant decrease in the R, and R eventually decreases to 0 at the confidence of 0.98 for all categories of objects. For the dominant small cars, it also decreases to 0 at the confidence of more than 0.9, indicating that the learned YOLO has a high recognition success for small cars. For large vehicles, the recall rate decreases to 0 at the confidence of more than 0.75, which is caused by too few samples and inconspicuous aerial photo features of large vehicles, but YOLO still has a high recognition accuracy.
(2) As R increases, the number of objects of a certain category that are predicted increases; i.e., those with a lower probability are gradually predicted, which shows that the YOLO model is fairly effective in recognizing vehicles from aerial videos. The model overall reaches the maximum F1 value of 0.86 at the confidence of 0.516, balancing the PR relationship very well. The PR plot and F1 value together reflect that the training has quite stable results and high accuracy.
(3) The loss in both the training and validation sets decreases significantly with the increase of training epoch, and the loss function reaches a stable and low level after 70 training epochs. The mAP@0.5 and mAP@[0.5:0.95] plots also reflect stability at over 70 epochs, exceeding 90% and 40%, respectively, and the extracted video frames also show good recognition results. The actual detection results show that the YOLOv5 recognition algorithm reprogrammed in this study is very accurate and can be well applied in vehicle detection.
(4) The trained YOLOv5 target detection algorithm replaces the original Faster R-CNN detector in DeepSORT, improving the detection accuracy and efficiency; the coordinate transformation calculations are performed between the UAV video of DJI Mavic 2 Pro and the actual road, to convert UAV video pixel coordinates to real-world spatial scales. Vehicle trajectory, speed and other elements are extracted, and the vehicle trajectory lines are smoothed to construct a more realistic model, avoiding the impact of “jagged” trajectories on the maneuverability of the driver model.

The collected track and speed information can effectively reflect the judgment of most drivers and extreme drivers in the face of specific road design and can be collected in large quantities, eliminating the inconvenience and inaccuracy of hiring drivers or conducting simulation experiments. In this study, the UAV video is shot at an angle completely perpendicular to the ground, so that the video images are not stretched and distorted, which makes it easy to perform coordinate conversion calculations and track extraction work. The problem with such shooting is that due to the altitude restriction, ramps with a radius larger than 60 m may not be fully included in the frame; if the whole ramps are included, the flight altitude may not only exceed the safe altitude, but also lead to the shooting of the vehicle being too small, which may result in the bounding boxes of the YOLOv5 being unable to detect the vehicle. The solution can be to adopt a photography method with a certain slope that needs to re-calculate the coordinate conversion; besides, the roads and vehicles will be stretched, which makes it difficult to locate each point of ramps. Meanwhile, the track drawing will not be stretched with the ramps and vehicles, which may lead to inaccurate positioning of the track and so on. All the above problems need to be studied in depth.

## Figures and Tables

**Table 1:** Pros and cons of different versions of YOLO algorithm comparison.

Version	Improvements	Pros	Cons	References
YOLOv1	Transforms the detection task into mathematical regression problems	High real-time detection rate	High positioning error and low recall rate	[42,43,44]
YOLOv2	Batch normalization; fine-tuning of classification models using high-resolution images; use of prior frames	Higher accuracy, provides flexibility, helps models make predictions in a variety of dimensions	More model parameters and poor performance on small-sized targets	[43,44]
YOLOv3	Adjusted network structure; multi-scale features used for target detection; soft-max replaced by logistic for target classification	Focuses on correcting positioning errors and optimizing detection efficiency, especially for smaller objects	Occupies more storage space and requires more initialization dataset samples and parameters	[44,45]
YOLOv4	Enhances training capabilities on traditional GPUs and combines many functions	Achieves optimal efficiency in target detection and higher accuracy	Complex target detection network structure, multiple parameters, high training configuration requirements	[42,44]
YOLOv5	Updated SACK training and center loss functions, enhanced career distinction	Reduced memory and computational costs, higher detection accuracy	Common branching of classification and regression tasks in YOLOv5 head hurts the training process	[42,46]
YOLOv6	Introduces a complex quantization strategy, implements post-training quantization and channel distillation	Reduced delays while maintaining accuracy, mitigates additional delay expenses	Increased parameter computation and computational cost	[44,47]
YOLOv7	Uses an auxiliary head training method and a tandem-based architecture	Finer-grained identification capability and higher detection accuracy	Slower detection speed	[43,44,48]
YOLOv8	Introduces anchorless models to independently handle objectivity, classification, and regression tasks	Has a lightweight module and high performance	Algorithms contain more convolutional layers and parameters with slower training speed	[44,47,48]
YOLOv9	Introduces programmable gradient information (PGI) with generic GELAN	Improved performance, enhanced lightweight modules	Lower detection accuracy, slower speed, requires a large amount of data	[49]
YOLOv10	NMS-free training, enables holistic modeling	Improved performance, reduced delays, and huge reductions in parameters and FLOPs	Lower detection accuracy, low speed, requires a large amount of data, small targets cannot be detected	[50]

**Table 2:** Hard/software version configuration for this study.

Name	Parameter or Version	Note	Company Name and Address
GPU memory	12 G	Hardware	NVIDIA Corporation, Santa Clara, CA, USA
RAM	16 G	Hardware	Generic, N/A
Disk memory	78 G	Hardware	Generic, N/A
Python	3.7.12	Software	Python Software Foundation, Wilmington, DE, USA
PyTorch	1.8.0	Software	Meta Platforms, Inc., Menlo Park, CA, USA
CUDA	11.2	Software	NVIDIA Corporation, Santa Clara, CA, USA
cudnn	8.1.0	Software	NVIDIA Corporation, Santa Clara, CA, USA

**Table 3:** Key specifications of DJI Mavic 2 Pro.

Parameter	Value
Camera model	Hasselblad L1D-20c
Focal length	10 mm
Aperture	f/2.8
Camera sensor	1-inch CMOS(13.2 mm × 8.8 mm)
Image resolution	4 K: 3840 × 2160; 2.7 K: 2688 × 1512; FHD: 1920 × 1080

**Table 4:** Relationship between relative flying height and shooting coverage for aerial photography.

Shooting Mode	Relative Flying Height (m)	GSD (cm)	Shooting Length (m)	Shooting Width (m)	Shooting Area (m2)
4K	100	Length: 4.64789, Width: 4.07408	132	88	11616
4K	150	Length: 6.97184, Width: 6.11112	198	132	26136
4K	200	Length: 9.29578, Width: 8.14816	264	176	46464
2.7K	100	Length: 4.91072, Width: 5.82011	132	88	11616
2.7K	150	Length: 7.36608, Width: 8.73017	198	132	26136
2.7K	200	Length: 9.82144, Width: 11.6402	264	176	46464
FHD	100	Length: 6.87500, Width: 8.14815	132	88	11616
FHD	150	Length: 10.3125, Width: 12.2223	198	132	26136
FHD	200	Length: 13.7500, Width: 16.2963	264	176	46464

## Data Availability

Data is contained within the article.
